# Efficacy of a Toothpaste Based on Microcrystalline Hydroxyapatite on Children with Hypersensitivity Caused by MIH: A Randomised Controlled Trial

**DOI:** 10.3290/j.ohpd.b2403649

**Published:** 2021-12-08

**Authors:** Vicky Ehlers, Ann Katrin Reuter, Evan-Bengü Kehl, Joachim Enax, Frederic Meyer, Jennifer Schlecht, Irene Schmidtmann, James Deschner

**Affiliations:** a Associate Professor, Department of Periodontology and Operative Dentistry, University Medical Centre of the Johannes Gutenberg University, Mainz, Germany. Conceived and designed the study, performed the study, conducted the data acquisition, analysed the data, wrote the paper, reviewed the manuscript, approved the manuscript prior to submission.; b Assistant Professor, Department of Periodontology and Operative Dentistry, University Medical Centre of the Johannes Gutenberg University, Mainz, Germany. Performed the study, conducted the data acquisition, reviewed the manuscript, approved the manuscript prior to submission.; c Student, Department of Periodontology and Operative Dentistry, University Medical Centre of the Johannes Gutenberg University, Mainz, Germany. Conducted the data acquisition, reviewed the manuscript, approved the manuscript prior to submission.; d Senior Scientist Oral Care, Research Department, Dr. Kurt Wolff GmbH & Co. KG, Bielefeld, Germany. Conceived and designed the study, reviewed the manuscript, approved the manuscript prior to submission.; e Senior Scientist Oral Care, Research Department, Dr. Kurt Wolff GmbH & Co. KG, Bielefeld, Germany. Conceived and designed the study, reviewed the manuscript, approved the manuscript prior to submission.; f Statistician, Institute of Medical Biostatistics, Epidemiology and Informatics (IMBEI), University Medical Centre of the Johannes Gutenberg University, Mainz, Germany. Analysed the data, wrote the paper, reviewed the manuscript, approved the manuscript prior to submission.; g Statistician, Institute of Medical Biostatistics, Epidemiology and Informatics (IMBEI), University Medical Centre of the Johannes Gutenberg University, Mainz, Germany. Conceived and designed the study, analysed the data, wrote the paper, reviewed the manuscript, approved the manuscript prior to submission.; h Professor and Chair, Department of Periodontology and Operative Dentistry, University Medical Centre of the Johannes Gutenberg University, Mainz, Germany. Conceived and designed the study, reviewed the manuscript, approved the manuscript prior to submission.

**Keywords:** children, hydroxyapatite, hypersensitivity, fluoride, molar incisor hypomineralisation (MIH)

## Abstract

**Purpose:**

Hypersensitivity is a frequent complaint in children with molar incisor hypomineralisation (MIH). This double-blind randomised controlled trial aimed to evaluate non-inferiority in hypersensitivity relief of a toothpaste containing microcrystalline hydroxyapatite compared to amine fluoride in children with MIH.

**Materials and Methods:**

Children were randomised into 2 groups: either hydroxyapatite (intervention) or amine fluoride toothpaste (control). The primary endpoint was pain sensation in response to tactile stimulus (Wong-Baker FACES Pain Rating Scale) 56 days after randomisation and analysed by mixed effects linear regression analysis. Non-inferiority was inferred if the upper limit of the one-sided 95% confidence interval (CI) of the difference between intervention and control group was below the non-inferiority margin of 1 in the ITT (intention-to-treat) and PP (per protocol) population.

**Results:**

Twenty-one children were randomised and 14 children finished the study per protocol. In the ITT population, hydroxyapatite was non-inferior to amine fluoride (mean difference: -0.75 95%CI [-∞;0.49]). In the PP population, non-inferiority could not be shown (-0.62 [-∞;1.08]).

**Conclusions:**

Overall non-inferiority in hypersensitivity relief of a toothpaste containing hydroxyapatite compared to amine fluoride could not be shown. However, the hydroxyapatite group tended to be less hypersensitive in both populations. Attrition of the PP population due to the COVID-19 pandemic led to loss of statistical power.

Molar incisor hypomineralisation (MIH) is regarded as a global disease with a worldwide prevalence of 2% to 40%.^32^ MIH seems to have a multifactorial pathogenesis; it is suggested that it can be caused by systemic conditions, environmental toxins or common childhood illnesses.^1,15,17,52^ Up to the present, the exact aetiology is still unknown.

In MIH enamel, the protein content, especially albumin, is increased, which results in an overall reduction of minerals in MIH-affected teeth.^19,20^ This leads to the conclusion that the mineralisation process of those teeth is incomplete during tooth development.^52^ MIH occurs in mild to severe forms.^42^ Clinically, those teeth have demarcated opacities between cream/white and yellow/brown colour with or without post-eruptive enamel breakdown.^18^ MIH-affected teeth can be scored and classified according to various indices.^11,25,41,54^ Mathu-Muju and Wright^41^ classified MIH into three severity levels with mild, moderate and severe MIH. The MIH treatment-need index (MIH-TNI) introduced by Steffen et al^54^ is based both on the extent of destruction of the tooth structure and the presence or absence of hypersensitivity. After diagnosis of MIH, treatment options must consider age, compliance and caries risk of the patient, type and extension of demarcated lesions, and hypersensitivity.^26^ Treatment modalities range from prevention of post-eruptive enamel breakdown and caries, management of hypersensitivity and pain, to restorative therapy or extraction with orthodontic treatment.^17,39^

Children with MIH-affected teeth frequently describe hypersensitivity and pain in those teeth.^2,49^ The affected teeth are more prone to post-eruptive enamel breakdown, which leads to dentin exposure.^2^ The enhanced hypersensitivity can lead to inadequate oral hygiene, thus increasing the susceptibility to developing caries.^5^ Consequently, the therapeutic strategy needs to reduce hypersensitivity to ensure adequate oral hygiene and to improve quality of life.^2,16,22^ In addition to post-eruptive enamel breakdown and hypersensitivity, there are other reported clinical problems for patients with MIH, resulting in treatment challenges for dentists: local anaesthesia problems, behavioural management problems, aesthetic problems of incisors, and loss of molars.^2,24,33,39^

Most commonly used and recommended are fluoride toothpastes for daily oral care.^38^ In a study by Bekes et al,^8^ the combined use of a desensitising toothpaste and mouthwash with arginine, calcium carbonate and fluoride was evaluated to reduce MIH-induced hypersensitivities. In the participating children, hypersensitivity was statistically significantly reduced during this 8-week trial.

Additional calcium and phosphate sources are also recommended for children with MIH.^2^ Amorphous calcium phosphates stabilised with milk proteins (casein), applied as casein phosphopeptide and amorphous calcium phosphate (CPP-ACP) in oral products, have been shown to remineralise MIH^6^ and statistically significantly reduce tooth sensitivity in children with MIH.^46^ However, casein is not vegan, and might lead to irritations in persons sensitive to milk proteins.^29^ Moreover, CPP-ACP does not minimise initial bacterial colonisation on enamel and dentin.^27^ In contrast to this, hydroxyapatite, Ca_5_(PO_4_)_3_(OH), can reduce bacterial biofilm formation and can be used without milk proteins.^28,35^ A hydroxyapatite-containing toothpaste achieved an efficacy comparable with an amine fluoride (500 ppm F^-^) toothpaste in remineralising initial caries and preventing demineralisation in primary teeth in situ.^3^ In a recent clinical trial, it was shown that a toothpaste with microcrystalline hydroxyapatite was not inferior to an amine fluoride toothpaste (500 ppm F^-^) on enamel caries progression in the primary dentition.^47^ This calcium phosphate is an effective agent used in toothpastes to prevent dentin hypersensitivity.^30,31,55,57^ Hydroxyapatite particles may promote enamel surface repair by forming a protective coating^37^ and act as a calcium and phosphate reservoir.^14^

The aim of our clinical trial was to evaluate non-inferiority of a toothpaste based on microcrystalline hydroxyapatite compared to a toothpaste based on amine fluoride with respect to hypersensitivity relief of MIH-affected teeth in children.

## Materials and Methods

### Study Design and Population

This monocentre, randomised, double-blind, active-controlled clinical trial evaluated non-inferiority of a microcrystalline hydroxyapatite toothpaste vs an amine fluoride toothpaste on reduction of hypersensitivity in MIH-affected molars. It was approved by the Ethics Committee of the state Rhineland-Palatinate, Germany (No. 2019-14558) and was registered in the German register for clinical trials (registration number: DRKS00020359, date of first registration: 19/12/2019). All methods were performed in accordance with relevant guidelines and regulations. The guidelines of the Declaration of Helsinki were observed. Written informed consent form was signed by children and parents prior to their participation in the study. From January 2020 to October 2020, patients were recruited from the Department of Periodontology and Operative Dentistry as well as the Department of Orthodontics at the University Medical Centre of the Johannes Gutenberg University, Mainz, Germany. In addition, children were recruited from paediatric dental offices in Mainz, Germany. For study participants, four appointments (T_0_ = screening; T_1_ = randomisation, baseline; T_2_ = 28±3 days after randomisation; T_3_ = 56±3 days after randomisation) were scheduled at the Department of Periodontology and Operative Dentistry, University Medical Centre of the Johannes Gutenberg University, Mainz, Germany. All participants who were randomised belonged to the intention-to-treat (ITT) population. Study participants with protocol violations (four or more days earlier/later than the scheduled visits or five or more missing entries in the toothbrushing diary) were excluded from the per protocol (PP) population.

### Inclusion and Exclusion Criteria

Inclusion criteria: age: 6-16 years; gender: female and male; presence of at least one hypersensitive, MIH-affected molar with response to a tactile stimulus scoring > 0 on the Wong-Baker FACES Pain Rating Scale (WBFS), and response to an air-blast stimulus scoring of 2 or 3 on the Schiff Cold Air Sensitivity Scale (SCASS).

Exclusion criteria: no MIH; MIH-affected molar with no hypersensitivity response to a tactile stimulus as a scored pain intensity of 0 on the WBFS or response to an air-blast stimulus as a defined score of 0 or 1 on the SCASS.

### Toothpastes and Toothbrushes

Both toothpastes (hydroxyapatite and amine fluoride) were provided in neutral plastic tubes of identical colour and shape. They carried a randomisation number; the toothpaste type associated with the randomisation number was known only to the Research Department, Dr. Kurt Wolff GmbH & Co. KG, Bielefeld, Germany.

The intervention toothpaste with 10% microcrystalline hydroxyapatite was a commercially available product (Kinder Karex Zahnpasta, Dr. Kurt Wolff; Bielefeld, Germany) and contained the following ingredients: aqua, hydrogenated starch hydrolysate, hydrated silica, hydroxyapatite, xylitol, silica, cellulose gum, aroma, 1,2-hexanediol, caprylyl glycol, sodium methyl cocoyl taurate, sodium sulfate, sodium cocoyl glycinate, and limonene.

The control toothpaste with amine fluoride (1400 ppm F^-^) was also a commercially available product (Elmex Junior Zahnpasta, CP GABA; Hamburg, Germany) and contained the following ingredients: aqua, hydrated silica, sorbitol, hydroxyethylcellulose, olaflur, aroma, saccharin, and limonene.

In addition to the assigned toothpaste, the study participants were also provided with a standardised electric toothbrush (Braun Oral-B PRO 600, P&G; Schwalbach, Germany) and with electric toothbrush heads for sensitive teeth (Braun Oral-B Sensi electric toothbrush heads, P&G). Participating children were instructed to brush their teeth with the assigned toothpaste, the provided toothbrush, and the brush head for 2 min in the morning and 2 min in the evening (i.e. 2x daily) over the whole observation period of 8 weeks. A toothbrushing diary was used to control toothbrushing frequency. During the study period, the children were instructed to use no other toothpastes and/or other dental care products, such as mouthwashes or gels. Furthermore, the study participants were instructed to refrain from eating and drinking for at least 1 h prior to clinical examinations. At the end of the study, children/parents were informed that they could then reconvene their routine oral hygiene with their preferred dental care products.

### Blinding and Randomisation

A randomisation list was generated by one of the statisticians using an SAS program (v 9.4; Cary, NC, USA). This list was sent to the Research Department, Dr. Kurt Wolff GmbH & Co. KG, where the toothpastes were packaged accordingly in neutral tubes. Neither dentists nor the analysing statistician (while writing the programs) were aware of the allocation. Intervention toothpaste (hydroxyapatite) and control toothpaste (amine fluoride) were handed out to the study participants by a study nurse, who was not involved in the clinical assessment of the study parameters. Randomisation was stratified by age at baseline: stratum A: age 6–11 years; stratum B: age 12–16 years.

### Instruments

Up to four MIH-affected molars per child were included as study teeth. Each study tooth was evaluated with two stimuli to assess tactile and air-blast hypersensitivity. For the tactile stimulus, a dental probe was applied. The pain intensity was scored on the WBFS, which ranges from 0 = no hurt, through 10 = hurts worst.^58^ For the air-blast stimulus, the air was delivered from an air syringe of a dental unit. The SCASS is defined according to Schiff et al.^51^ Oral hygiene was evaluated with the approximal plaque index (API) in %, as described by Lange et al.^36^ For assessment of API, plaque disclosing solution was used (Mira-2-Ton, Hager & Werken; Duisburg, Germany).

Participants were asked about the toothpaste taste and general evaluation of the toothpaste, both measured on a VAS ranging from 0 to 10 (0 = very bad; 10 = very good), and further use of toothpaste was answered with yes or no.

Study teeth were scored according to the MIH Treatment Need Index (MIH TNI) as described by Steffen et al.^54^ Dental examinations included recording decayed, missing, filled teeth/surfaces in the primary dentition (dmft/dmfs), and decayed, missing, filled teeth/surfaces in the secondary dentition (DMFT/DMFS). Table 1 indicates each instrument and during which study visits it was collected.

**Table 1:** Data collection during the course of the study

	Instrument	T_0_ (screening, study consent)	T_1_ (baseline, randomisation)	T_2_ (follow-up 28 ± 3 days)	T_3_ (follow-up 56 ± 3 days)
Sociodemographic characteristics (sex, age)		X			
MIH classification	MIH-TNI	X			
Dental assessment	dmf-t/s, DMF-T/S	X			
Toothpaste, toothbrush, toothbrush heads			Handing out		Collect
Toothbrushing diary			Handing out	Control	Collect
Pain sensation in response to tactile stimulus	WBFS	X	X	X^2^	X^1^
Pain sensation in response to air-blast stimulus	SCASS	X	X	X^2^	X^2^
Oral hygiene	API		X	X^2^	X^2^
Rating of taste	VAS				X^2^
General rating	VAS				X^2^
Intention of further use					X^2^

^1^ Primary endpoint; ^2^ secondary endpoint. API; approximal plaque index; DMF-T/S: decayed, missing, filled teeth/surfaces in the secondary dentition; dmf-t/s: decayed, missing, filled teeth/surfaces in the primary dentition; MIH: molar incisor hypomineralisation; MIH-TNI: MIH treatment need index; SCASS: Schiff cold air sensitivity scale; VAS: visual analogue scale; WBFS: Wong-Baker FACES Pain Rating Scale.

### Primary Endpoint and Secondary Endpoints

The primary endpoint was pain sensation in response to tactile stimulus, measured on a WBFS ranging from 0 to 10 at 56 (± 3) days after randomisation. Secondary endpoints were pain sensation in response to tactile stimulus measured on the same WFBS at 28 (± 3) days, pain sensation in response to air-blast stimuli measured by SCASS at 28 (± 3) days and 56 (± 3) days, API at 28 (± 3) days and 56 (± 3) days, taste of toothpaste and general evaluation of the toothpaste, both measured on a VAS ranging from 0 to 10, and further use of toothpaste.

### Sample Size Calculation

Sample size was calculated for the primary endpoint. The non-inferiority margin was set at ε = 1 and a standard deviation (SD) of 1.2 was assumed in both arms. With 20 patients per group, one tooth included per patient, non-inferiority could be demonstrated at the 5% level with 80% power. A total of 40 patients were planned to be accrued, using 1:1 randomisation to treatment or control.

### Statistical Analysis

The descriptive analysis included a comparison of baseline characteristics as well as primary and secondary endpoints between intervention and control group. Variables were summarised by appropriate statistics. For categorical variables, absolute and relative frequencies (in %) were reported. For continuous variables, means and standard deviations (SD) were reported for participant characteristics, means and 95% confidence intervals (CI) obtained from linear mixed regression models were reported for study tooth characteristics.

The primary endpoint was analysed by a linear mixed regression model in both the ITT and the PP population. Non-inferiority was inferred if the upper limit of the one-sided 95% CI of the difference between intervention and control groups was below the non-inferiority margin of ε = 1. Overall non-inferiority in hypersensitivity relief was concluded if the intervention group was non-inferior to the control group in both the ITT and the PP population. Results were reported by mean differences with 95% CI, and the p-value was deduced from the linear mixed regression model.

The secondary endpoint, pain sensation in response to tactile stimulus, at T_2_ was analysed in the same manner as the primary endpoint. Pain sensation in response to air-blast stimuli at T_2_ and T_3_ was analysed using a two-sided Fisher’s exact test. If more than one study tooth could be included per child, one tooth was randomly selected. Oral hygiene at T_2_ and T_3_ was analysed using a one-sided two-sample t-test. Rating of taste and general rating of the toothpaste at T_3_ were analysed with the one-sided Wilcoxon rank-sum test. Intention of further use of the toothpaste at T_3_ was analysed by a two-sided Fisher’s exact test. All secondary endpoints were analysed in both the ITT and the PP population and were exploratory. Results were reported by mean differences with 95% CI where applicable (deduced from the linear mixed regression model) and p-value.

Missing primary and secondary endpoints at T_3_ were filled by T_2_ values if possible (last observation carried forward, LOCF). In case more than 20% of primary or secondary endpoints was missing (after LOCF, if applicable), data were imputed 5 times using multivariate imputation by chained equations and 50 iterations (R package mice).^56^ Only pooled results were then reported. All analyses were carried out with R version 4.0.1 (R Foundation; Vienna, Austria).^48^

## Results

### Subjects

**Fig 1:**
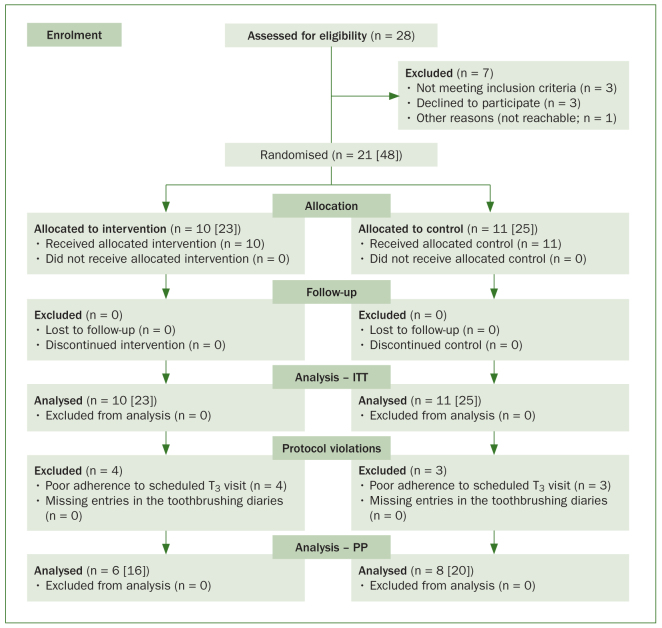
Flow chart of the study participants. ITT: intention-to-treat; PP: per protocol.

Of 28 subjects screened at T_0_, 21 subjects (48 study teeth) were randomised to use either a toothpaste containing hydroxyapatite (n = 10 [23 study teeth]; intervention group) or toothpaste containing amine fluoride (n = 11 [25 study teeth]; control group). The ITT population therefore included 21 subjects with 48 study teeth. The PP population was reduced to 14 subjects (36 study teeth) due to poor adherence to scheduled visits at T_3_ (Fig 1); 3 and 4 participants came four or more days too early or too late for their appointment, respectively.

Most participants were female (71%) and all were under 12 years old. Mean ± SD oral hygiene measured by API was 62.6% ± 23.9%. A total of 33% of children had a dmf-t/dmf-s (primary dention) score over 0, and 52% of children had a DMF-T/DMF-S (secondary dention) score above 0. In 62% of the participants, more than one study tooth could be included in the study. Most study teeth had enamel breakdown (69%). At baseline, mean (95%CI) pain sensation in response to tactile stimulus was 5.3 (4.3–6.4) and the majority of study teeth were grade 2 on the SCASS scale (65%). A comparison between intervention and control group regarding sociodemographic and clinical characteristics is shown in Tables 2 and 3.

**Table 2:** Demographic and oral health characteristics of included participants (ITT population, participant level)

		Intervention group (n = 10)^1^	Control group (n = 11)^1^
**Gender**			
Male	n (%)	4 (40)	2 (18)
Female	n (%)	6 (60)	9 (82)
**Age (years, T_1_)**	mean ± SD	8.6 ± 1.5	8.4 ± 1.2
6–11	n (%)	10 (100)	11 (100)
12–16	n (%)	0 (0)	0 (0)
**API (T_1_)**	mean ± SD	68.3 ± 22.2	57.3 ± 25.2
**dmf-t (T_0_)**			
0	n (%)	5 (50)	9 (82)
>0	n (%)	5 (50)	2 (18)
**dmf-s (T_0_)**			
0	n (%)	5 (50)	9 (82)
>0	n (%)	5 (50)	2 (18)
**DMF-T (T_0_)**			
0	n (%)	5 (50)	5 (45)
>0	n (%)	5 (50)	6 (55)
**DMF-S (T_0_)**			
0	n (%)	5 (50)	5 (45)
>0	n (%)	5 (50)	6 (55)
**Included study teeth per participant**			
1 tooth	n (%)	5 (50)	3 (27)
2 teeth	n (%)	0 (0)	4 (36)
3 teeth	n (%)	2 (20)	2 (18)
4 teeth	n (%)	3 (30)	2 (18)

There were no missing values in baseline characteristics. ^1^Number relates to included participants. API: approximal plaque index; DMF-T/S: decayed, missing, filled teeth/surfaces in the secondary dentition; dmf-t/s: decayed, missing, filled teeth/surfaces in the primary dentition; SD: standard deviation.

**Table 3:** Pain, discomfort, and hypersensitivity of included study teeth (ITT population, study tooth level)

		Intervention group (n = 23)^1^	Control group (n = 25)^1^
**MIH-TNI (T_0_)**			
Index 3	n (%)	6 (26)	9 (36)
Index 4a	n (%)	7 (30)	6 (24)
Index 4b	n (%)	10 (43)	9 (36)
Index 4c	n (%)	0 (0)	1 (4)
**Pain sensation in response to tactile stimulus: WBFS**^2^ **(T**_1_**)**	mean [95%CI]	5.6 [4.0-7.1]	5.1 [3.6-6.6]
**Pain sensation in response to air-blast stimulus: SCASS (T**_1_ **)**			
Grade 1	n (%)	2 (9)	4 (16)
Grade 2	n (%)	14 (61)	17 (68)
Grade 3	n (%)	7 (30)	4 (16)

There were no missing values in baseline characteristics. ^1^ Number relates to included study teeth. ^2^ Range: 0-10 cm. CI: confidence intervals; MIH: molar incisor hypomineralisation; MIH-TNI: MIH treatment need index; SCASS: Schiff cold air sensitivity scale; WBFS: Wong-Baker FACES Pain Rating Scale.

### Primary Endpoint, Pain sensation in Response to Tactile Stimulus at T_3_

**Fig 2:**
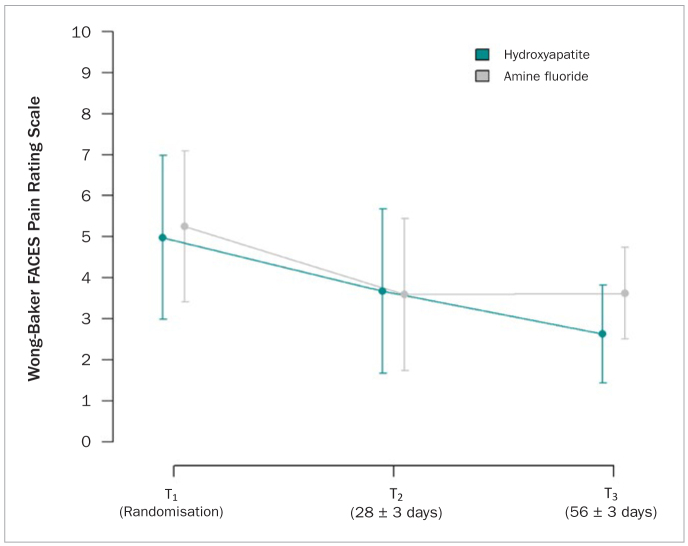
Pain sensation in response to tactile stimulus over time. Pain sensation was measured on a Wong-Baker FACES Pain Rating Scale ranging from 0 (no hurt) through 10 (hurts worst). Patients were randomised to use a toothpaste containing hydroxyapatite or amine fluoride. Means with 95% confidence intervals were deduced from linear mixed regression models.

In the ITT population, mean pain sensation in response to tactile stimulus at T_3_ was on average 2.6 (1.5–3.7) in the intervention and 3.4 (2.4–4.4) in the control group. The mean difference (95% CI) between intervention and control group was -0.75 (-∞; 0.49) (p = 0.013). Therefore, the intervention group was non-inferior to the control group regarding hypersensitivity relief in the ITT population (Table 4). Figure 2 shows the trend in pain sensation in response to tactile stimulus over the whole study period.

**Table 4:** Results of primary and secondary endpoint analyses (ITT and PP population, study tooth level)

		ITT population				PP population			
		Intervention group (n = 23)^1^	Control group (n = 25)^1^	p	Statistic*	Intervention group (n = 16)^1^	Control group (n = 20)^1^	p	Statistic*
**Primary endpoint**									
Pain sensation in response to tactile stimulus: WBFS^2^ (T_3_)	mean [95%CI]	2.6 [1.5–3.7]	3.4 [2.4–4.4]	0.013	3	2.6 [0.9–4.3]	3.1 [1.7–4.5]	0.058	3, 5
Missing	n	4	4			4	4		
**Secondary endpoints**									
Pain sensation in response to tactile stimulus: WBFS^2^ (T_2_)	mean [95%CI]	3.7 [1.7–5.7]	3.6 [1.7–5.4]	0.311	3, 5	3.7 [0.4–7.0]	3.7 [0.8–6.6]	0.341	3, 5
Missing	n	5	6			4	6		
Pain sensation in response to air-blast stimulus: SCASS (T_3_)				0.735	4			0.757	4, 5
Grade 0	n (%)	0 (0)	0 (0)			0 (0)	0 (0)		
Grade 1	n (%)	14 (78)	18 (86)			9 (75)	15 (94)		
Grade 2	n (%)	4 (22)	2 (10)			3 (25)	1 (6)		
Grade 3	n (%)	0 (0)	1 (5)			0 (0)	0 (0)		
Missing	n	5	4			4	4		
Pain sensation in response to air-blast stimulus: SCASS (T_2_)				0.697	4, 5			0.508	4, 5
Grade 0	n (%)	0 (0)	0 (0)			0 (0)	0 (0)		
Grade 1	n (%)	5 (28)	12 (63)			3 (25)	11 (79)		
Grade 2	n (%)	12 (67)	6 (32)			9 (75)	3 (21)		
Grade 3	n (%)	1 (6)	1 (5)			0 (0)	0 (0)		
Missing	n	5	6			4	6		

Missing values at T_3_ were imputed by T_2_ values (LOCF) where possible. Distribution relates to values after LOCF. ^1^Number relates to included study teeth. ^2^Range: 0-10 cm. 3*Linear mixed effects model, one-sided (non-inferiority margin = 1); 4*Fisher’s exact test, two-sided. If several study teeth per participant were included, one study tooth was randomly selected; 5*missing values (after LOCF, if applicable) were multiply imputed; CI: confidence intervals; ITT: intention-to-treat; LOCF: last observation carried forward; PP: per protocol; SCASS: Schiff cold air sensitivity scale; WBFS: Wong-Baker FACES Pain Rating Scale.

In the PP population, pain sensation in response to tactile stimulus was on average 2.6 (0.9-4.3) in the intervention and 3.1 (1.7-4.5) in the control group. The difference between intervention and control group was -0.62 95%CI (-∞; 1.08) (p = 0.058). Therefore, non-inferiority of the intervention group could not be shown in the PP population (Table 4).

### Secondary Endpoints

#### Pain sensation in response to tactile or air-blast stimuli

The mean difference of pain sensation in response to tactile stimulus at T_2_ between intervention and control study teeth was 0.38 (-∞; 2.52) (p = 0.311) in the ITT population. In the PP population, the mean difference between intervention and control group was 0.31 (-∞; 3.16) (p = 0.341) (Table 4).

In terms of air-blast stimuli, a total of 78% of the intervention and 86% of the control study teeth reached grade 1 on the SCASS scale at T_3_ in the ITT population (p = 0.735). In the PP population, 75% of the intervention and 94% of the control group reached grade 1 on the SCASS scale at T_3_ (p = 0.757) (Table 4).

Regarding pain sensation in response to air-blast stimulus at T_2_, a total of 28% of the intervention and 63% of the control study teeth reached grade 1 on the SCASS scale in the ITT population (p = 0.697). In the PP population, 25% of the intervention and 79% of the control group reached grade 1 on the SCASS scale at T_2_ (p = 0.508) (Table 4).

#### Oral hygiene

Looking at oral hygiene at T_3_, mean ± SD API was 47.4% ± 18.9% in the intervention and 51.3% ± 11.7% in the control group in the ITT population (p = 0.302). In the PP population, mean ± SD API at T_3_ was 37.6% ± 18.1% in the intervention group and 49.4% ± 11.8% in the control group (p = 0.233) (Table 5).

**Table 5:** Results of secondary endpoint analyses (ITT and PP population, participant level)

		ITT population				PP population			
		Intervention group (n = 10)^1^	Control group (n = 11)^1^	p	Statistic*	Intervention group (n = 6)^1^	Control group (n = 8)^1^	p	Statistic*
**Secondary endpoints**									
Oral hygiene: API (T_3_)	mean ± SD	47.4 ± 18.9	51.3 ± 11.7	0.302	3	37.6 ± 18.1	49.4 ± 11.8	0.233	3, 6
Missing	n	3	1			2	1		
Oral hygiene: API (T_2_)	mean ± SD	48.9 ± 12.4	46.2 ± 10.6	0.678	3, 6	51.8 ± 13.5	47.1 ± 12.0	0.680	3, 6
Missing	n	3	3			2	3		
Rating of taste: VAS^2^ (T_3_)	mean ± SD	7.4 ± 2.5	8.9 ± 1.7	0.909	4	6.8 ± 2.4	9.1 ± 1.2	0.917	4, 6
Missing	n	2	1			2	1		
General rating: VAS^2^ (T_3_)	mean ± SD	9.2 ± 0.9	8.2 ± 2.3	0.283	4	8.8 ± 1.0	8.1 ± 2.3	0.629	4, 6
Missing	n	2	1			2	1		
Intention of further use (T_3_)				1.000	5			N/A	5-7
Yes	n (%)	8 (100)	9 (90)			4 (100)	7 (100)		
No	n (%)	0 (0)	1 (10)			0 (0)	0 (0)		
Missing	n	2	1			2	1		

Missing values for API at T_3_ were imputed by T_2_ values (LOCF) where possible. Distribution relates to values after LOCF. ^1^Number relates to included participants; ^2^Range: 0-10 cm; 3*Independent samples t-test, one-sided; 4*Wilcoxon rank-sum test, one-sided; 5*Fisher’s exact test, two-sided; 6*missing values (after LOCF, if applicable) were multiply imputed; 7*Fisher’s exact test was not possible as all participants indicated intention to use further. API: approximal plaque index; ITT: intention-to-treat; LOCF: last observation carried forward; PP: per protocol; SD: standard deviation; VAS: visual analogue scale.

At T_2_, mean ± SD API was 48.9% ± 12.4% in the intervention and 46.2% ± 10.6% in the control group in the ITT population (p = 0.678). In the PP population, mean ± SD API at T_2_ was 51.9% ± 13.5% in the intervention group and 47.1% ± 12.0% in the control group (p = 0.68) (Table 5).

#### Taste rating

At T_3_, mean ± SD taste rating was 7.4 ± 2.5 in the intervention and 8.9 ± 1.7 in the control group in the ITT population (p = 0.909). In the PP population, mean ± SD rating of taste was 6.8 ± 2.4 in the intervention group and 9.1 ± 1.2 in the control group (p = 0.917) (Table 5).

#### General rating at T_3_

The mean ± SD general rating was 9.2 ±0.9 in the intervention and 8.2 ± 2.3 in the control group in the ITT population (p = 0.283). In the PP population, mean ± SD general rating was 8.8 ±1.0 in the intervention group and 8.1 ± 2.3 in the control group (p = 0.629) (Table 5).

#### Further use intention at T_3_

In the ITT population, all participants in the intervention and 90% of the participants in the control group intended to keep using the toothpaste (p = 1.000). In the PP population, all participants in the intervention and in the control group intended to keep using the toothpaste (test not possible) (Table 5).

### Safety

No serious adverse events were reported in the course of the study. In total, only one adverse event (AE) was reported in 21 subjects: 0 AE were found in subjects who used the intervention toothpaste and 1 AE was found among the subjects who used the control toothpaste.

## Discussion

Non-inferiority in hypersensitivity relief of a toothpaste containing hydroxyapatite compared to amine fluoride after 8 weeks was shown in the ITT population. In the PP population, non-inferiority could not be shown. Consequently, we were not able to show overall non-inferiority in hypersensitivity relief. However, due to the COVID-19 pandemic and resulting lockdowns, we were not able to recruit 40 patients as planned and some participants could not keep their appointments on time (four or more days earlier/later than the scheduled visits). This led to an underpowered comparison, especially in the PP population (n = 14). On a descriptive basis, we observed that children in the hydroxyapatite group seemed to be less hypersensitive in both the ITT and PP populations, compared to children in the amine fluoride group. Concerning secondary endpoints, we were not able to show any differences between the hydroxyapatite and amine fluoride groups. The descriptive analysis indicated that the difference in pain sensation in response to tactile stimulus seemed to develop later than 28 days after randomisation. More children in the control group reached grade 1 on the SCASS scale, especially at T_2_. Oral hygiene was comparable in the intervention and control groups. The taste of the control toothpaste was rated slightly better than the intervention toothpaste in both the ITT and PP populations. However, the general rating of the toothpaste was slightly in favour of the intervention toothpaste. All children in the intervention group intended to keep using the toothpaste.

More girls than boys seemed to be afflicted with hypersensitive MIH molars in both the intervention and the control groups. In a study by Ozgül et al,^45^ girls exhibited statistically significantly higher sensitivity than boys, and the authors concluded that gender is an important factor in the sensitivity of MIH teeth. Although results from a study by Pasini et al^46^ showed that females report slightly greater sensitivity compared to males, no statistically significant difference was observed when comparing females with males. Apart from hypersensitivity, it was found that MIH itself equally affects boys and girls,^23^ and no statistically significant difference between males and females in a literature research including 70 studies was reported.^59^ All of the study participants were between 6 and 11 years old, and none of them were in the age range of 12 to 16 years old. This is in accordance with Zhao et al,^59^ who found that the prevalence of MIH among children 10 years or younger was much higher than that among older children.

The novel coronavirus disease (COVID-19) pandemic and resulting lockdowns influenced this trial. Worldwide, many medical as well as dental clinics and surgeries reduced treatment of patients, with only to urgent and non-deferrable care being provided.^4^ In Austria at the beginning of the pandemic, 78.6% of paediatric dentists and members of the Austrian Society of Paediatric Dentistry only offered emergency services.^10^ In a survey from Brazil, it was shown that most parents (66.6%) would only seek urgent dental care for their children and only 17.8% of the parents were willing to take their children to dental care regardless of the treatment.^12^ Recruitment for this study started in January 2020, shortly before the World Health Organisation (WHO) characterised COVID-19 as a pandemic in March 2020. Originally, it was planned to recruit 40 children, which is in agreement with the study by Pasini et al.^46^ However, due to the COVID-19 pandemic and resulting lockdowns, only 28 children could be recruited. A total of 21 children participated in the present study and 48 MIH-affected hypersensitive molars were included, which is comparable to the study by Bekes et al,^8^ where 19 children with 56 MIH-affected teeth were enrolled.

All teeth included in this trial were subjected to tactile and air-blast stimuli. Both stimuli are widely recommended to assess dentin hypersensitivity in patients, and both are physiological, encountered in everyday life and are easily controlled.^8,50^ These stimuli have been applied to hypersensitive MIH-affected teeth in children in previous studies.^8,46^ For pain-severity assessment, the visual analogue scale is a common method in adults, but in children, scales based on faces were used.^8^ For children, such face-scales have become the most popular method to measure children’s self-reports of pain and a majority of children prefer to use face-scales.^13,34^ Because of the paediatric population in this study, the WBFS^58^ was used.

Oral hygiene of the study participants was merely fair in both the intervention and the control groups at baseline, with an API of 68.3% (intervention group) and 57.3% (control group). It improved during the study, but was comparable in both treatment groups. All participating children had hypersensitive teeth, which can lead to compromised oral hygiene. It was demonstrated in a study by Ebel et al^16^ that the efficacy of oral hygiene in children with MIH decreases with increasing hypersensitivity.

The efficacy of hydroxyapatite in toothpastes on dentin hypersensitivity has been evaluated in various clinical studies with adult patients and different observation periods, from 3 days up to 8 weeks.^43,44,55,57^ However, the efficacy of hydroxyapatite-containing toothpaste on children with hypersensitivity caused by MIH has not yet been investigated.

Especially in the early posteruptive period, some MIH-affected first permanent molars exhibit high sensitivity.^21^ In the past, although little or no research evidence existed, various desensitising agents were regarded to be of value in the management of sensitivity in MIH-affected teeth.^21^ In the preventive approach to MIH, fluoride-containing toothpastes, fluoride varnishes and CPP-ACP products might be useful for MIH-patients and have more recently been proposed to be helpful in reducing sensitivity; however, further research is required.^39^ A review concluded that only a limited number of mainly observational studies exists which investigated treatment options for MIH.^17^ Concerning MIH-affected molars, non-invasive and invasive/restorative treatment options are available, and the indication for different treatment options depends on the severity of MIH and hypersensitivity.^17^ In enhanced prevention, remineralisation and sensitivity management of MIH, beside fluoridated toothpaste, CPP-ACP products (the most common being Tooth Mousse and MI Paste Plus) can be recommended. Moreover, products containing both fluoride and ACP (such as Enamelon Preventive Treatment Gel) as well as a toothpaste containing calcium sodium phosphosilicate (NovaMin), can be used to reduce sensitivity.^2^ Further treatment options for molars with MIH consist of resin infiltration; restoration using glass-ionomer cement (GIC), resin-modified GIC and resin composite; full- or partial-coverage restorations, e.g. preformed metal crowns, preformed malleable composite crowns, indirect onlay; and extraction of severely affected molars.^2^

At present, only a few studies have focussed on remineralisation of MIH teeth. In a study by Baroni and Macchioni,^6^ an improvement in the enamel morphology of 30 MIH molars was seen after the use of CPP-ACP. A study by Ozgül et al^45^ with MIH-affected incisors evaluated the effect of desensitising agents (fluoride, CPP-ACP, and CPP-ACP with fluoride) applied with and without ozone therapy. The tested desensitising agents effectively reduced hypersensitivity. CPP-ACP was more effective, and ozone use prolonged the effect of CPP-ACP.

To the best of our knowledge, only two studies exist which focus on hypersensitivity treatment in MIH-affected molars of children.^8,46^ The clinical trial by Bekes et al^8^ was the first to evaluate development and management of hypersensitivity in MIH children and the first to describe a treatment with arginine-containing products. The tested toothpaste and mouthwash contained arginine, calcium carbonate and fluoride. However, no control groups were included, neither an active nor a negative control. In contrast, in the present study, an active control group was included, which agrees with Pasini et al.^46^ Hypersensitivity assessment was performed with air-blast and tactile stimuli, which conforms with both Bekes et al^8^ and Pasini et al.^46^ The time points of hypersensitivity assessment were at baseline, immediately after treatment and after one, two, four and eight weeks in the study by Bekes et al.^8^ In another study, two time points were chosen: at baseline and after 120 days.^46^ In the present study, pain assessments were conducted at baseline, after 28 days and after 56 days. In the study by Bekes et al,^8^ the mean tactile hypersensitivity score measured at the baseline examination was 2.1, after four weeks 0.8 and after eight weeks 0.6, whereas in the present study, pain sensations upon tactile stimulus were higher at baseline, with 5.6 in the intervention group and 5.1 in the control group. After 28 days, pain sensation upon tactile stimulus was 3.7 (intervention group) and 3.6 (control group), and after 56 days, it decreased further to 2.6 (intervention group) and 3.4 (control group). However, the low pain levels after four and eight weeks described by Bekes et al^8^ could have not been achieved in this trial. In comparison with the study by Pasini et al,^46^ where CPP-ACP in the test group and fluoride toothpaste in the control group were examined, sensitivities to tactile stimulation were higher at baseline, with 7.8 in the test group and 7.5 in the control group, than in the present trial. The sensitivity to tactile stimulus was reduced after 120 days to 3.8 (test group) and 7.2 (control group), which is still higher than in our study, although the observation period was not as long in the study by Pasini et al.^46^

Recently, some studies or protocols have been published concerning sealing technique, desensitising agents and the use of laser in MIH-affected molars.^7,9,40,53^ In a pilot study with 12 children, the efficacy in hypersensitivity relief of MIH-affected molars using two sealing techniques (composite sealant Clinpro Sealant in combination with Scotchbond Universal and glass ionomer Ketac Universal) in a split-mouth design was investigated.^9^ Clinical pain assessments were performed with SCASS and VAS before and immediately after treatment, as well as after one, four, eight and 12 weeks. A total of 24 molars with SCASS 2 or 3 were included. The application of the sealant statistically significantly decreased hypersensitivity immediately after treatment and thereafter at all time points. No statistically significant difference between the two tested materials was observed; both sealing techniques were successful in reducing hypersensitivity. A recent study by Bekes et al^7^ is the first to evaluate changes in oral health-related quality of life (ORHQoL) before and after sealing in children with hypersensitive MIH-affected molars. Sealing led to a statistically significant improvement of OHRQoL immediately after treatment and throughout the 12-week study. In a case report with an 8-year-old boy with MIH, the use of a high-power laser (Nd:YAG laser) followed by application of a desensitising agent (Gluma Desensitizer) on the first mandibular molars was described.^40^ Pain assessment was performed with air sensitivity test (VAS) before and immediately after treatment as well as after one week and one month. The authors concluded that the use of laser and desensitising agent for treatment of dentin hypersensitivity caused by MIH was effective in reducing the pain level. A protocol has been published for a future study^53^ including 140 adult patients (age between 18 and 35 years) with at least one tooth with MIH and with sensitivity ≥ 4 on VAS. The participants will be divided into four groups: control group (placebo), sealant group (treatment with PermaSeal), low-level laser (LLL) group and LLL and sealant group. The follow-up to evaluate dentin hypersensitivity is planned immediately after treatment, after one week, one month, three and six months.

One of the limitations of the present trial is the lack of a placebo group as a negative control. For ethical reasons, no negative control was planned for this study. Other limitations include the small sample size due to the COVID-19 pandemic and resulting lockdowns, which led to an underpowered comparison between the two toothpastes. Especially in children, hypersensitivity assessment with air-blast and tactile stimulus has a subjective nature. With the knowledge of participating in a study, study participants might progressively improve their oral hygiene, which could have a positive effect on hypersensitivity relief. Moreover, none of the study participants were personally related to the investigator, thus, compliance bias could not have influenced their responses.

Further research is needed to evaluate the efficacy of the intervention toothpaste with a larger sample size and a longer follow-up period to confirm the results of this study and to show non-inferiority in both the ITT and PP population.

As hypersensitivity is regarded as a common condition, the findings of this trial can be generalised for all children suffering from hypersensitivity.

## Conclusions

In the present clinical trial of children with hypersensitive MIH-affected molars, it was shown that both toothpastes (hydroxyapatite versus amine fluoride) were effective in relieving hypersensitivity and maintaining desensitisation for 8 weeks. In terms of hypersensitivity relief, overall non-inferiority of the hydroxyapatite compared to the amine fluoride containing toothpaste could not be shown. Studies with a larger sample sizes and longer follow-up periods may still be needed for further evaluation of non-inferiority of a hydroxyapatite containing toothpaste. However, in both populations (ITT and PP), children in the hydroxyapatite group tended to show less hypersensitivity.
